# Elevated endothelial dysfunction-related biomarker levels indicate the severity and predict sepsis incidence

**DOI:** 10.1038/s41598-022-26623-y

**Published:** 2022-12-19

**Authors:** Gaosheng Zhou, Jingjing Liu, Hongmin Zhang, Xiaoting Wang, Dawei Liu

**Affiliations:** Department of Critical Care Medicine, Peking Union Medical College Hospital, Chinese Academy of Medical Sciences and Peking Union Medical College, 1# Shuai Fu Yuan, Dong Cheng District, Beijing, 100730 China

**Keywords:** Biomarkers, Medical research

## Abstract

This study was conducted to investigate the relationship between serum endothelial dysfunction-related biomarker levels and organ dysfunction severity in septic patients and the predictive value of these levels during sepsis. In total, 105 patients admitted to the Department of Critical Care Medicine were enrolled between September 2020 and November 2021. Serum syndecan-1 and soluble thrombomodulin(sTM) levels were measured by enzyme-linked immunosorbent assay, and clinical and laboratory data were recorded. Enroll patients were divided into the infection (n = 28), septic nonshock (n = 31), and septic shock (n = 46) groups . Serum syndecan-1 (102.84 ± 16.53 vs. 55.38 ± 12.34 ng/ml), and sTM(6.60 ± 1.44 ng/ml vs. 5.23 ± 1.23 ng/ml, P < 0.01) levels were increased in the septic group compared with those in the infection group. Serum syndecan-1 levels were closely positively correlated with serum sTM (r_s_ = 0.712, r^2^ = 0.507, P < 0.001). Additionally, serum syndecan-1(r_s_ = 0.687, r^2^ = 0.472, P < 0.001) and sTM levels (r_s_ = 0.6, r^2^ = 0.36, P < 0.01) levels were significantly positively correlated with the sequential organ failure assessment scores respectively. Syndecan-1 (AUC 0.95 ± 0.02, P < 0.0001) was more valuable for prediction sepsis than was sTM (AUC 0.87 ± 0.04, P < 0.0001). Compared with sTM (AUC 0.88 ± 0.03, P < 0.001), syndecan-1 (AUC 0.95 ± 0.02, P < 0.001) and SOFA score (AUC 0.95 ± 0.02, P < 0.001) were better predictors of septic shock. Serum syndecan-1 and sTM levels were associated with organ dysfunction severity in septic patients, and both were good predictors for early identification of sepsis, particularly in patients undergoing septic shock.

## Introduction

Sepsis is a life-threatening organ dysfunction caused by a dysregulated host response to infection and is associated with significant morbidity and mortality^1^. The pathogenetic mechanism of sepsis is highly complex, and endothelial dysfunction is an important factor leading to organ dysfunction^2^. Endothelial cells shift toward a proinflammatory, proadhesive, and procoagulant phenotype during sepsis. Moreover, the endothelial cells' functional modifications are initially adaptive but ultimately become harmful leading to multiorgan dysfunction during sepsis^3^.

The glycocalyx is the constituent of the endothelial surface layer, which regulates vascular permeability, adhesion of leukocytes and platelets, shear stress, and inflammatory processes^4^.

Syndecan-1 is a member of the polysaccharide syndecan family, which belongs to the family of transmembrane heparan sulfate proteins in the glycocalyx, and circulating syndecan-1 is a marker of endothelial glycocalyx degradation^5^. Previous studies have shown that syndecan-1, is shed and released into the bloodstream during severe infection, reflecting glycocalyx damage and hence a superficial endothelial disruption^6,7^. Studies have demonstrated that syndecan-1 shedding is associated with both sepsis presence and severity^8,9^. Thrombomodulin (TM), an integral endothelial cell membrane protein, is critical in maintaining vascular thrombus resistance^10^. TM has anticoagulant activity as well as anti-inflammatory and cytoprotective effects^11^. Previous studies have shown that TM is enzymatically cleaved from endothelial cells and released into the bloodstream under direct endothelial cell damage, thereby reflecting profound endothelial dysfunction^12^.

As the innermost layer of the arterial wall, the syndecan-1 is the first protein to be affected^13^. However, soluble thrombomodulin (sTM) is derived from direct damage to endothelial cells, not through secretion, which appears later in the circulating. Syndecan-1 and sTM serve as markers of endothelial cell injury in humans but to varying degrees. Furthermore, circulating syndecan 1 and sTM levels have been independently associated with mortality in patients with both trauma and acute myocardial infarction^14,15^. However, limited data exist regarding the relationship between syndecan-1, sTM, and organ dysfunction severity and their predictive value in sepsis. This study was conducted to investigate the relationship between syndecan-1, sTM, and organ dysfunction severity in septic patients, and the predictive value of these biomarkers during sepsis.

## Methods

### Participants

ThE study was conducted at the Department of Critical Care Medicine between September 2020 and November 2021, approved by the hospital institutional review board (Ethics Approval No. JS-3283) and performed following the Declaration of Helsinki. All enrolled patients or their families provided written informed consent. Patients were screened for enrolment within the first 24 h of admission.

Patients were divided into the infection, septic nonshock, and septic shock groups. The infection group comprised patients admitted to the intensive care unit (ICU) for an active infection. The septic nonshock group comprised patients with an acute change in Sequential Organ Failure Assessment (SOFA) score ≥ 2 points consequent to the infection and for whom septic shock was ruled out. The septic shock group comprised patients requiring the administration of vasopressors and had lactate levels > 2 mmol/l on the day of ICU admission^1^. Sepsis/septic shock was diagnosed according to The Third International Consensus Definitions for Sepsis and Septic Shock (Sepsis 3.0)^1^. We excluded patients who were aged < 18 years, had ICU stays of < 24 h, had massive bleeding or pulmonary embolism, had a heart attack or acute exacerbation of previous heart disease in the previous week, had heart surgery in the prior week or lacked informed consent.

### Data collection

Baseline clinical and laboratory data were collected within 24 h after ICU admission, including patient age, sex, hemodynamic parameters, blood chemistry, SOFA scores, and Acute Physiology and Chronic Health Evaluation (APACHE) II scores.

### Blood sample collection

Peripheral blood samples were collected within the first 24 h of ICU admission and immediately centrifuged. Serum was separated by centrifugation at 2500×*g* for 15 min and stored immediately at −80 °C until assessment by enzyme-linked immunosorbent assay (ELISA).

### ELISA measurements

The soluble biomarkers, serum syndecan-1 and sTM, established biomarkers of endothelial glycocalyx and cell injury, respectively, were measured using commercially available immunoassays, the Human Syndecan-1 ELISA kit (CUSABIO, Catalog No. CSB-E1498h, Wu Han, China) and Human soluble Thrombomodulin ELISA kit (Abcam, Catalog No. ab214029, Cambridge, England).

### Statistical analysis

Normally distributed data are expressed as the mean and standard deviation and were compared using Student’s t-test or one-way analysis of variance. Non-normally distributed data are presented as the median and interquartile intervals and were compared using the Mann–Whitney U test. Categorical variables were compared using the chi-square test and are recorded as proportions. Correlations between normally distributed data were analyzed using the Pearson method. Spearman correlation analysis was performed on non-normally distributed data. Statistical analysis was performed using SPSS 23 (SPSS, IBM, Armonk, NY, USA) and GraphPad Prism 8.0 (GraphPad Software Inc., San Diego, CA, USA). Two-tailed P values ≤ 0.05 were considered statistically significant. Sample size was estimated based on a priori power calculation indicating an 80% power to detect differences in syndecan-1 and soluble thrombomodulin(sTM) (effect size 0.7) among groups at a 0.05 significance level using a power and sample size website.

### Ethics approval and consent to participate

The study was approved by the PUMCH institutional review board (Ethics Approval No.JS-3283). All subjects provided written informed consent.

## Results

### Patients’ general and clinical characteristics

**Figure 1:**
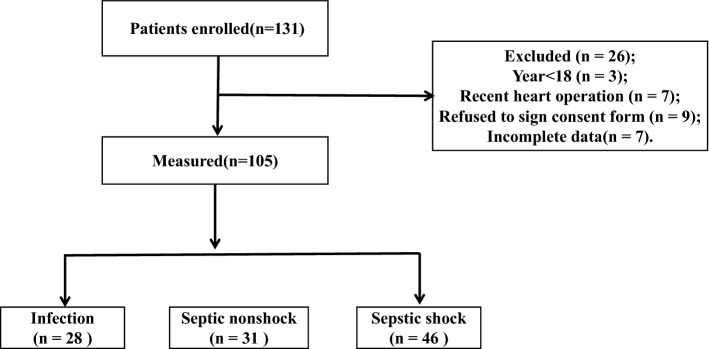
Screening flowchart of patients.

We screened 131 patients for enrolment, and 105 were included in this study (Fig. 1). The patients were divided into the infection (n = 28), septic nonshock (n = 31), and septic shock (n = 46) groups. Age, sex, venous-to-arterial carbon dioxide difference (Pv-aCO2), central venous blood oxygen saturation (ScvO_2_), mean arterial blood pressure (MAP), and central venous pressure (CVP) did not significantly differ among the groups. Patients with septic shock had significantly higher SOFA scores and lactate levels than did the septic nonshock group. Table 1 shows the basic characteristics of all enrolled patients.

**Table 1:** Patient characteristics at ICU admission.

Categories	Infection (n = 28)	Septic non-shock (n = 31)	Septic shock (n = 46)	P value
Age (years, mean ± SD)	62 ± 18	59 ± 18	62 ± 17	0.768
Gender (male, %)	64.29	77.42	63.04	0.591
APACHEII score	15 (11–16)	16 (14–19)	17 (15–19)*	0.013
SOFA score	0 (0–2)	5 (4–8)*	11 (9–14)*^#^	0.000
HR (bpm)	96 ± 24	106 ± 20	110 ± 22*	0.031
MAP (mmHg)	89 ± 12	91 ± 16	84 ± 15	0.070
CVP (mmHg)	–	11 ± 7	8 ± 4	0.327
P_v-a_CO_2_ (mmHg)	–	4 (1.7–6.9)	5.1 (2.7–6.1)	0.453
ScvO_2_ (%)	–	77.3 ± 9.3	75.2 ± 10.3	0.716
Lactate (mmol/l)	1.0 (0.8–1.6)	1.6 (1.1–2.3)	4.0 (2.2–7.8)*^#^	0.000

Data was presented by mean standard deviation, n (%), or median (interquartile range). For normally distributed data, comparisons among multiple groups were conducted by one-way analysis of variance, and those between two groups were carried out with the Least Significant Difference (LSD) post hoc test.

For data without normal distribution, Post hoc tests and between-group analyses were performed by Mann–Whitney nonparametric tests and Bonferroni corrected.

*APACHE* Acute Physiology and Chronic Health Evaluation, *SOFA* sequential organ failure assessment score, *HR* heart rate, *MAP* mean arterial blood pressure, *CVP* central venous pressure, *P*_*v-a*_*CO*_*2*_ venous-to-arterial carbon dioxide difference (Pv-aCO2), *ScvO*_*2*_ central venous blood oxygen saturation.

*P < 0.05 for the comparison between infection and septic non-shock, between infection and septic shock.

^#^P < 0.05 for the comparison between septic non-shock and septic shock.

### Serum levels of endothelial damage-related biomarkers in a derivation cohort

**Figure 2:**
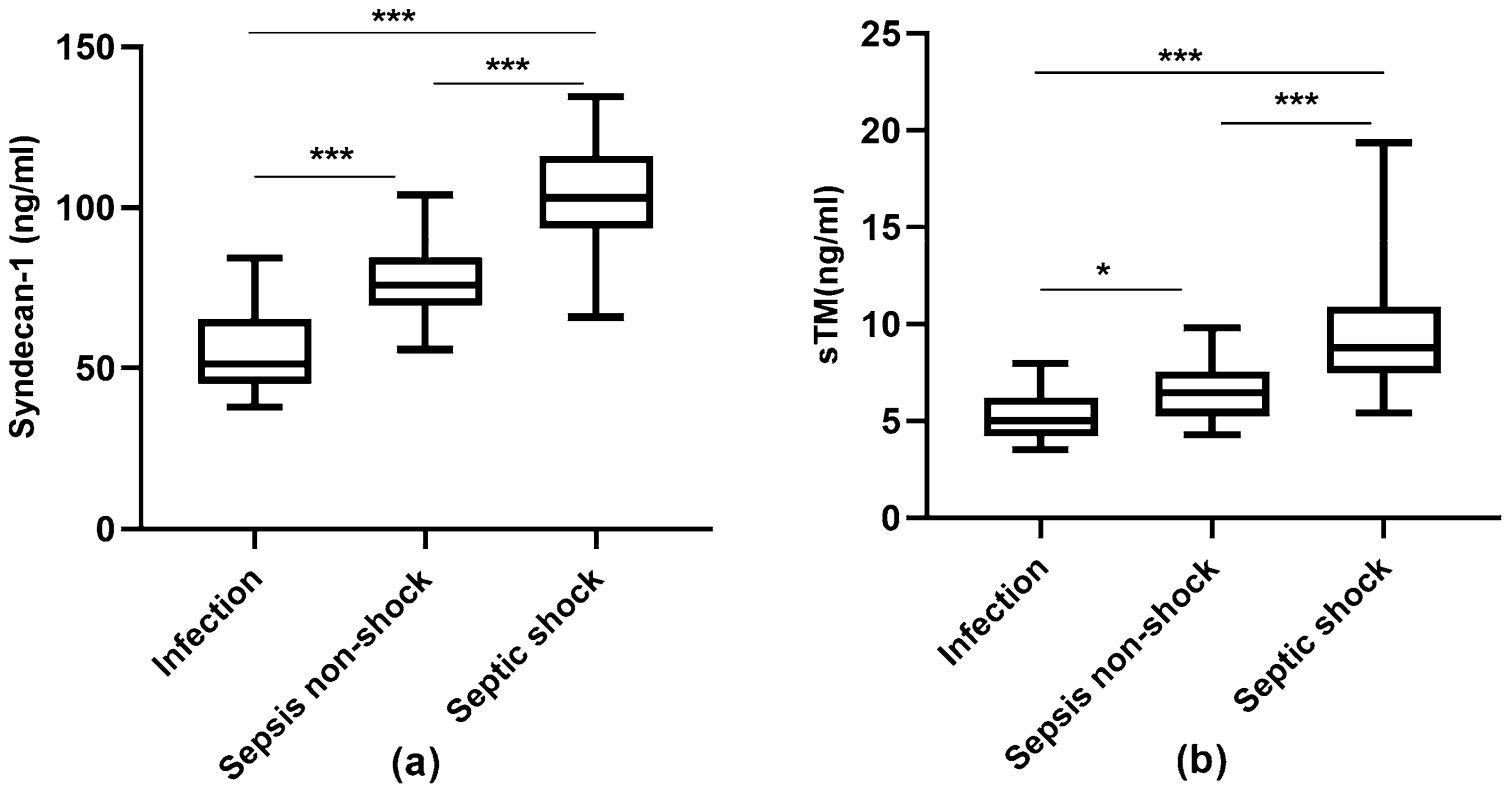
Comparison of serum syndecan-1 levels (**a**) and sTM (**b**) levels among groups***. ***Comparisons among multiple groups were conducted by one-way analysis of variance, and those between two groups were carried out with the Least Significant Difference (LSD) post hoc test. P < 0.05 were considered statistically significant. *Denotes P < 0.05, ***Denotes P < 0.001. *sTM* serum thrombomodulin.

Figure 2 and Table 2 show the changes in endothelial damage-related biomarkers in the progression from infection to septic shock. Serum syndecan-1 and sTM levels increased progressively from infected patients to septic nonstock patients to septic shock patients. The syndecan-1 and sTM level variation tendencies were similar among the groups. Serum syndecan-1 concentrations on ICU admission were significantly increased in the septic shock group and septic nonshock groups compared with those of the infection group.

**Table 2:** Clinical characteristics.

Categories	Infection (n = 28)	Septic non-shock (n = 31)	Septic shock (n = 46)	P value
Syndecan-1 (ng/ml)	55.38 ± 12.34	76.06 ± 10.51*	102.84 ± 16.53*^#^	0.000
sTM (ng/ml)	9.67 ± 3.38	6.60 ± 1.44*	5.23 ± 1.23*^#^	0.000
OI (mmHg)	327 ± 112	320 ± 144	296 ± 134	0.561
TB (μmol/l)	13.7 (12.3–28.7)	13.5 (9.4–21.9)	17.8 (12.7–31.0)	0.093
Cr (μmol/l)	72 (32–109)	82 (58–156)	90 (82–196)	0.152
Fibrinogen (g/l)	4.2 ± 1.9	4.3 ± 2.0	3.9 ± 4.7	0.889
Dimer (mg/l)	3.7 (2.6–9.6)	4.3 (2.4–13.1)	5.4 (2.8–9.9)	0.058
PT (s)	14.6 (12.6–16.1)	13.9 (12.9–16.0)	15.4 (14.2–20.0)	0.049
APTT (s)	30.7 (27.4–35.2)	31.6 (28.5–36.8)	30.0 (26.3–39.2)	0.896
INR (ratio)	1.3 (1.2–1.5)	1.1 (1.1–1.5)	1.3 (1.2–1.4)^#^	0.009
Procalcitonin (ug/l)	1.1 (0.2–10.0)	1.4 (0.6–12.7)	8.0 (1.9–30.3)*	0.003
PLT (*10^9^)	196 ± 79	171 ± 99	136 ± 93*	0.022

Data was presented by mean standard deviation. For normally distributed data, comparisons among multiple groups were conducted by one-way analysis of variance, and those between two groups were carried out with the Least Significant Difference (LSD) post hoc test. For data without normal distribution, Post hoc tests and between-group analyses were performed by Mann–Whitney nonparametric tests and Bonferroni corrected.

*sTM* serum thrombomodulin, *OI* oxygen index, *TB* total bilirubin, *Cr* creatinine, *PT* prothrombin time, *APTT* activated partial thromboplastin time, *INR* International Normalized Ratio, *PLT* platelet.

*P < 0.05 for the comparison between infection and septic non-shock, between infection and septic shock.

^#^P < 0.05 for the comparison between septic non-shock and septic shock.

Syndecan-1 levels were significantly increased in the septic shock group (102.84 ± 16.53 ng/ml) compared with those of the septic nonshock (76.06 ± 10.51 ng/ml) and infection (55.38 ± 12.34 ng/ml) groups. Serum sTM concentrations were significantly increased in the septic shock and septic nonshock groups compared with those of the infection group.

The septic shock group had higher sTM levels than did the septic nonshock group (9.67 ± 3.38 ng/ml vs. 6.60 ± 1.44 ng/ml, P < 0.01), and patients with septic nonshock had higher sTM levels than did patients with infection (6.60 ± 1.44 ng/ml vs. 5.23 ± 1.23 ng/ml, P < 0.01).

However, as result shown, no statistical difference in the serum syndecan-1 and sTM was seen when all patients (including infection ,sepsis non-shock and septic shock patients) were stratified by bacteremia and non-bacteremia (syndecan-1 82.47(61.14,103.07) vs. 75.84(69.13,87.33) ng/ml), P = 0.924; sTM 6.75(5.26,8.70) vs.7.39(5.81,8.89) ng/ml, P = 0.711). Moreover, no significant difference was observed according to the identified pathogens (gram-positive vs. gram-negative) involved in all patients (syndecan-1 86.48(66.24,99.17) vs.79.67(59.14,103.98) ng/ml, P = 0.684; sTM 6.89(5.34,8.92) vs.6.68(5.23,8.65) ng/ml, P = 0.927).

### Serum syndecan-1 and sTM levels were associated with organ function and disease severity

To establish whether serum syndecan-1 and sTM levels could determine organ function and disease severity, we assessed the correlations between these levels and the SOFA and APACHE II scores on ICU admission.

**Figure 3:**
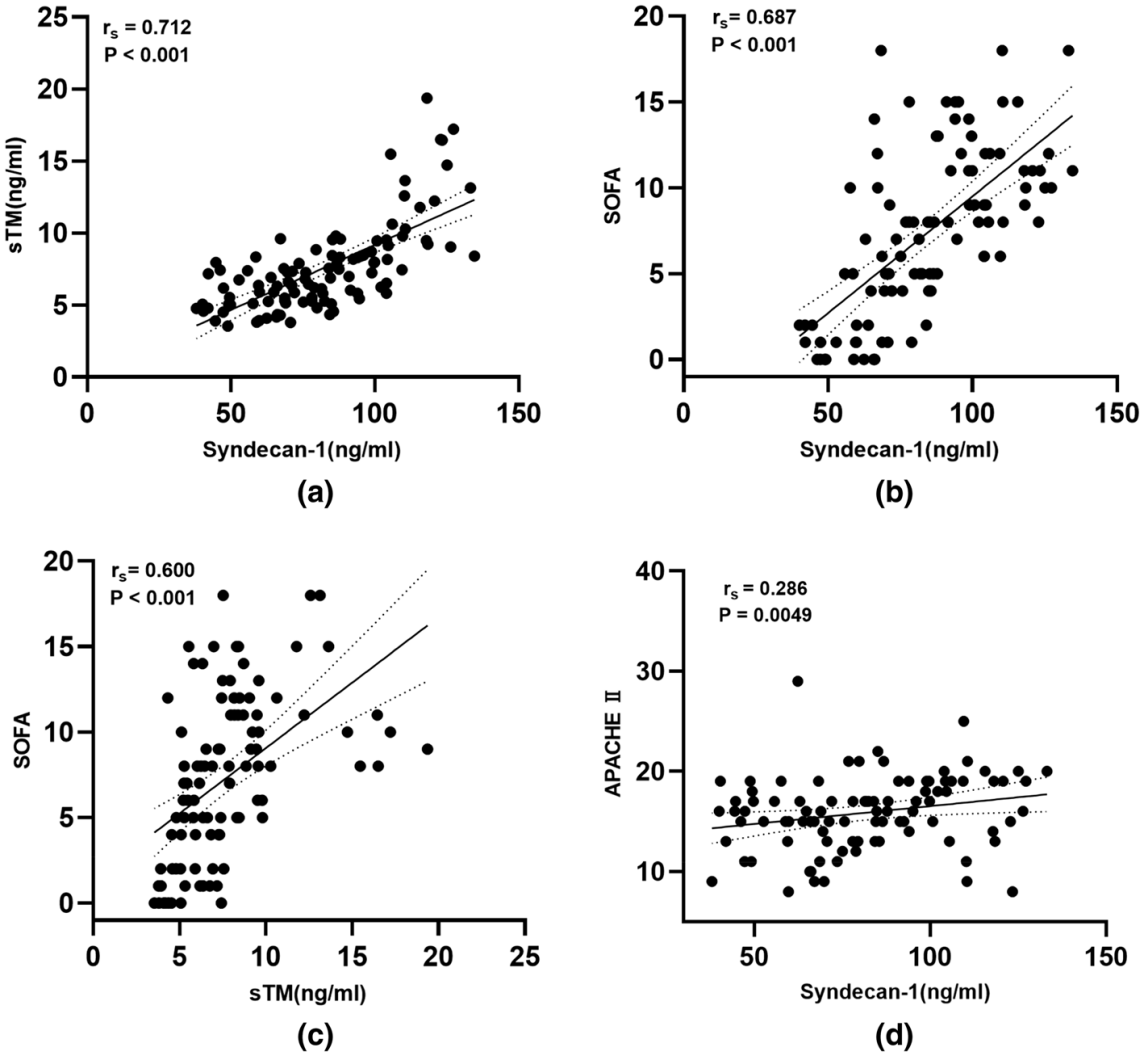
Correlation analysis. (**a**) Correlation between serum syndecan-1 and sTM in all the patients enrolled on ICU admission. (**b**) Correlation between serum syndecan-1 and SOFA score in all the patients enrolled on ICU admission. (**c**) Correlation between serum sTM and SOFA score in all the patients enrolled on ICU admission. (**d**) Correlation between serum syndecan-1 and APACHE II score in all the patients enrolled on ICU admission. P < 0.05 were considered statistically significant. *sTM* serum thrombomodulin, *SOFA* sequential organ failure assessment score, *ICU* intensive care unit.

Serum syndecan-1 levels were positively correlated with serum sTM (r_s_ = 0.712, r^2^ = 0.507, P < 0.001, Fig. 3a), the both serum syndecan-1 (r_s_ = 0.687, r^2^ = 0.472, P < 0.001, Fig. 3b) and sTM (r_s_ = 0.6, r^2^ = 0.36, P < 0.01, Fig. 3c) levels were positively correlated with SOFA scores respectively. Additionally, serum syndecan-1 levels were weakly associated with APACHE II score (r_s_ = 0.286, r^2^ = 0.082, P = 0.0049, Fig. 3d); however, we found no correlation between serum sTM levels and APACHE II score (r = 0.184, r^2^ = 0.034, P = 0.073).

We further assessed whether serum syndecan-1 and sTM levels were correlated with organ dysfunction at study enrollment and found that neither serum syndecan1 levels nor sTM levels were significantly correlated with oxygen index, total bilirubin, or creatinine (data not shown).

### Serum syndecan-1 and sTM levels were associated with tissue perfusion

**Figure 4:**
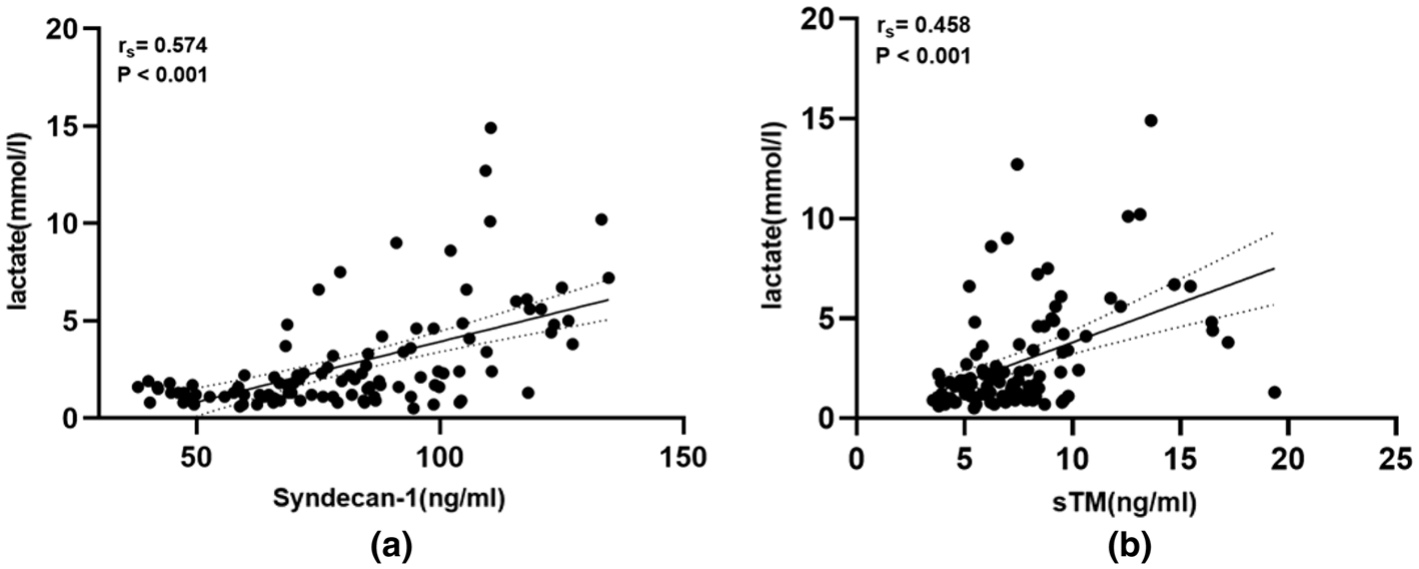
Serum syndecan-1 and sTM levels were associated with tissue perfusion. (**a**) Correlation between serum syndecan-1 and Lac in all the patients enrolled on ICU admission. (**b**) Correlation between serum sTM and Lac in all the patients enrolled on ICU admission. P < 0.05 were considered statistically significant. *sTM* serum thrombomodulin, *ICU* intensive care unit.

To assess the relationship between endothelial damage and tissue perfusion, we assessed the association between serum syndecan-1 and sTM levels and lactate (lac). Serum syndecan-1 (r_s_ = 0.574, r^2^ = 0.329, P < 0.001, Fig. 4a) and sTM (r_s_ = 0.458, r^2^ = 0.210, P < 0.001, Fig. 4b) levels in all patients admitted to the ICU were significantly positively correlated with lactate, demonstrating that endothelial damage was closely related to tissue perfusion(Fig. 4).

### Serum syndecan-1 was associated with coagulation function

Endothelial injury induces coagulation abnormalities during sepsis; therefore, we assessed the association between serum syndecan-1 and sTM levels and coagulation function parameters.

Correlation analysis showed that serum syndecan-1 levels were weakly correlated with prothrombin time (PT) (r_s_ = 0.286, r^2^ = 0.082, P = 0.003) and international normalized ratio (INR) (r_s_ = 0.337, r^2^ = 0.114, P = 0.0004) and not correlated with activated partial thromboplastin time (APTT) (r_s_ = 0.063, P = 0.526), D-dimer (r_s_ = 0.173, P = 0.087), or fibrinogen(Fib) (r_s_ = − 0.054, P = 0.584). Serum sTM levels were not correlated with PT (r_s_ = 0.096, P = 0.331), APTT (r_s_ = 0.038, P = 0.704), D-dimer (r_s_ = 0.040, P = 0.698), Fib (r_s_ = − 0.190, P = 0.053), or INR (r_s_ = 0.191, P = 0.051).

### Ability of endothelial damage biomarkers to predict the incidence of sepsis and septic shock

**Figure 5:**
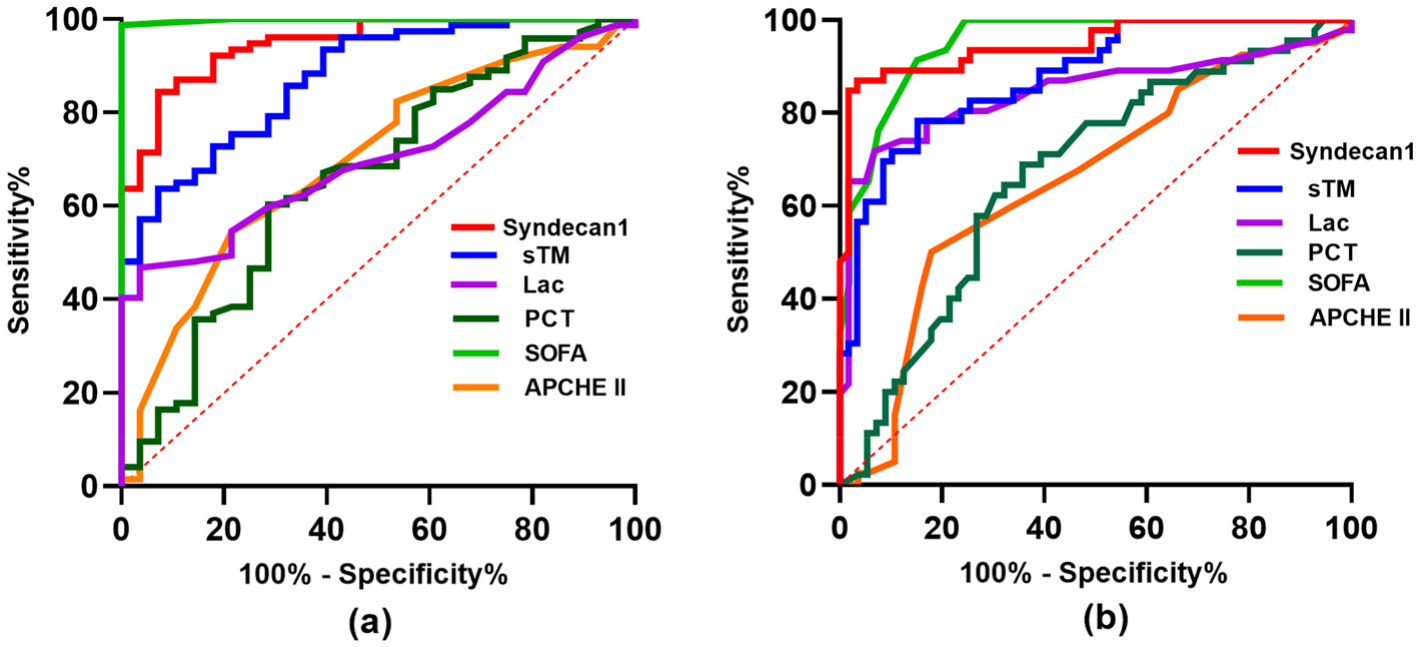
Receiver operating characteristic curve analysis. The ability of Syndecan1 and sTM on ICU admission to predict the incidence of sepsis (**a**) or septic shock (**b**). *sTM* serum thrombomodulin, *ICU* intensive care unit, *ICU* intensive care unit, *APACHE II score* Acute Physiology and Chronic Health Evaluation II score, *SOFA* sequential organ failure assessment score, *Lac* lactate, *PCT* Procalcitonin.

We calculated the cut-off value, sensitivity, and specificity of syndecan1 and sTM as predictors of sepsis or septic shock for the enrolled patients using the receiver operating characteristic curve(Tables 3, 4 and Fig. 5).

**Table 3:** Receiver operating characteristic (ROC) analysis for Syndecan1 and sTM in Sepsis.

	AUC ± SE	P value	Cut-off value	Sensitivity%	Specificity%
Syndecan1	0.95 ± 0.02	< 0.0001	70.91 ng/ml	84.42	92.86
sTM	0.87 ± 0.04	< 0.0001	7.21 ng/ml	63.64	92.86

*sTM* serum thrombomodulin.

**Table 4:** Receiver operating characteristic (ROC) analysis for Syndecan1 and sTM in Septic shock.

	AUC ± SE	P value	Cut-off value	Sensitivit%	Specificity%
Syndecan1	0.95 ± 0.02	< 0.001	87.14 ng/ml	86.96	96.61
sTM	0.88 ± 0.03	< 0.001	7.43 ng/ml	64.43	84.75

*sTM* serum thrombomodulin.

For diagnosing sepsis(Fig. 5a, Table 3), SOFA score was the best predictor (AUC 0.99 ± 0.001, P < 0.0001), and syndecan-1 (AUC 0.95 ± 0.02, P < 0.0001) was a better predictor than was sTM (AUC 0.87 ± 0.04, P < 0.0001), Procalcitonin (PCT) (AUC 0.66 ± 0.06, P = 0.0135), Lac (AUC 0.70 ± 0.05, P < 0.001), or APACHE II score (AUC 0.70 ± 0.06, P = 0.0025).

To diagnose septic shock, syndecan-1 (AUC 0.95 ± 0.02, P < 0.001) and SOFA score (AUC 0.95 ± 0.02, P < 0.001) were better predictors than were sTM (AUC 0.88 ± 0.03, P < 0.001), Lac (AUC 0.85 ± 0.04, P < 0.001), PCT (AUC 0.67 ± 0.05, P < 0.01), APACHE II score (AUC 0.65 ± 0.06, P = 0.01).

## Discussion

Our results indicated the following. First, both syndecan-1 and sTM levels were significantly increased in the septic shock group compared with those of the septic nonshock and the infection groups. The syndecan-1 and sTM levels gradually increased as the disease progressed, suggesting that endothelial injury was gradually aggravated. Syndecan-1 and sTM levels were significantly higher in patients with septic shock than in patients with infection and septic nonshock.

Moreover, serum syndecan-1 and sTM levels were elevated as the disease severity increased. Notably, serum syndecan-1 and sTM levels were positively correlated with both SOFA score and lactate. These results demonstrated the value of syndecan-1 and sTM levels as predictors of sepsis or septic shock incidence on the day of ICU admission.

Endothelial dysfunction is common in adult ICU patients, especially in critical patients with infection. Endothelial injury is a well-known hallmark of sepsis, but new data continue to emerge that further reveal the pathophysiology of endothelial damage in sepsis and its association with disease severity, including the applicability of biomarkers for outcomes^16,17^. However, different studies have yielded varying conclusions.

Anand et al. reported significant correlations between syndecan-1 levels and disease severity^18^. Deng et al. found that patients with sepsis had significantly higher serum sTM levels that were positively correlated with the disease severity^19^. Here, we revealed that the serum syndecan-1 and sTM levels were elevated with the disease severity, which is consistent with previous findings, but the molecular mechanism of this elevation remains undefined.

Syndecan-1 is a proteoglycan in the endothelial glycocalyx. Circulating syndecan-1 is a marker of endothelial glycocalyx degradation, which reflects superficial endothelial damage^20^. However, the mechanism underlying the endothelial glycocalyx during sepsis remains unclear. Metalloproteinases are activated in inflammatory states by reactive oxygen species and proinflammatory cytokines, which cleave proteoglycans directly from the endothelial cell membrane during sepsis^4^. The degraded glycocalyx layer leads to an increased permeability to plasma proteins and fluids, causing interstitial leakage. Several studies have demonstrated elevated syndecan-1 levels as a marker of glycocalyx degradation in patients with sepsis^21,22^.

Furthermore, endothelial dysfunction ultimately contributes to ending multiorgan damage during sepsis or septic shock. Considering the close relationship between endothelial function and organ function during sepsis^1^, we also explored the correlation between serum endothelial dysfunction biomarker levels and the SOFA scores and found that serum syndecan-1 and sTM levels were closely related to SOFA scores. Previous studies have shown that plasma syndecan-1 and sTM increased progressively and significantly across groups with increasing infectious severity and correlated significantly with organ failure as measured by SOFA scores in patients with varying degrees of infectious disease^23^.

Similarly, a large multicenter study of 1103 critically ill patients predominantly suffering from sepsis showed a strong association between epitheliopathy and organ failure^17^, demonstrating that patients with sepsis had higher plasma syndecan-1 and sTM levels than did non-infected patients. These results are consistent with the above idea and support our conclusion. Although the correlation was low in previous studies, our results showed a stronger correlation, which may be related to patient and sample heterogeneity and requires further confirmation.

Previous studies indicated that high syndecan-1 and sTM levels independently predicted liver and renal failure, respectively, and high sTM was further associated with an increased risk of developing multiple organ failure^24^. However, further analysis showed no significant correlations between syndecan-1, sTM, and organ function-related biomarker levels in septic patients. Moreover, endothelial dysfunction shifts toward a proapoptotic, proinflammatory, proadhesive, and procoagulant phenotype during sepsis, thus indicating the central role of endothelial dysfunction in the cross-talk during inflammation coagulation^25,26^. A previous study found an independent association between high circulating syndecan-1 levels and coagulopathy in a smaller cohort of 184 patients with severe sepsis or septic shock^23^.

Nevertheless, the correlations between endothelial dysfunction and coagulation found in the present study were relatively weak; thus, this correlation remains uncertain.

Sepsis-induced organ dysfunction is associated with inflammatory and coagulation responses in the endothelium as described above. Previous studies showed that endothelial dysfunction may result in decreased blood perfusion and therefore may aggravate lactic acidemia^27^. Microvascular obstruction and systemic endothelial dysfunction have been independently associated with plasma lactate in patients with falciparum malaria^28^. Here, we revealed that serum syndecan-1 and sTM levels were significantly correlated with lactate, which was consistent with previous findings, indicating that endothelial damage was closed correlated with tissue perfusion. Perfusion abnormalities caused by endothelial dysfunction may be another important cause of sepsis-related organ failure. Our previous study proposed mitochondrial dysfunction as an important cause of sepsis-related organ failure^29^. Thus, endothelial function induced by mitochondrial dysfunction might be an additional mechanism during sepsis.

Although clinicians know that endothelial activation and dysfunction play critical roles in the pathophysiology of sepsis and represent important therapeutic targets for reducing sepsis mortality, a large gap remains between basic research and clinical applications of endothelial activation and dysfunction. Thus, endothelial damage must be identified early in sepsis or septic shock. Elevated serum syndecan-1 and sTM levels could be used to alert clinicians of endothelial injury, and in clinical treatment, clinicians should put more emphasis on endothelial function and protecting endothelial cells from injury during sepsis.

### Limitations

This study has several limitations. First, our sample size was limited, and replication studies with a larger sample size are needed. Second, our findings were observational, hence, no causality can be inferred. Third, the results should be interpreted with caution owing to the heterogeneity of enrolled patients. Fourth, we analyzed only blood taken from the initial draw and did not follow the biomarker dynamics over time. Fifth, we included no healthy control group, and the serum syndecan-1 and sTM levels at baseline need to be measured.

## Conclusion

Syndecan-1 and sTM levels were positively associated with organ damage in patients with sepsis and septic shock. Serum syndecan-1 and sTM may be promising biomarkers for early diagnosis of sepsis, particularly for patients with septic shock.

## Data Availability

Data are available on request from the corresponding author.
